# Supplementary material for: An In Silico Design of a Vaccine against All Serotypes of the Dengue Virus Based on Virtual Screening of B-Cell and T-Cell Epitopes
Source: Biology (Basel). 2024 Aug 30;13(9):681. doi: 10.3390/biology13090681 (PMC11428656; doi:10.3390/biology13090681)
Supplement: Supplementary file 1 [file biology-13-00681-s001.zip › Supplementary File 2 Figures.pdf]

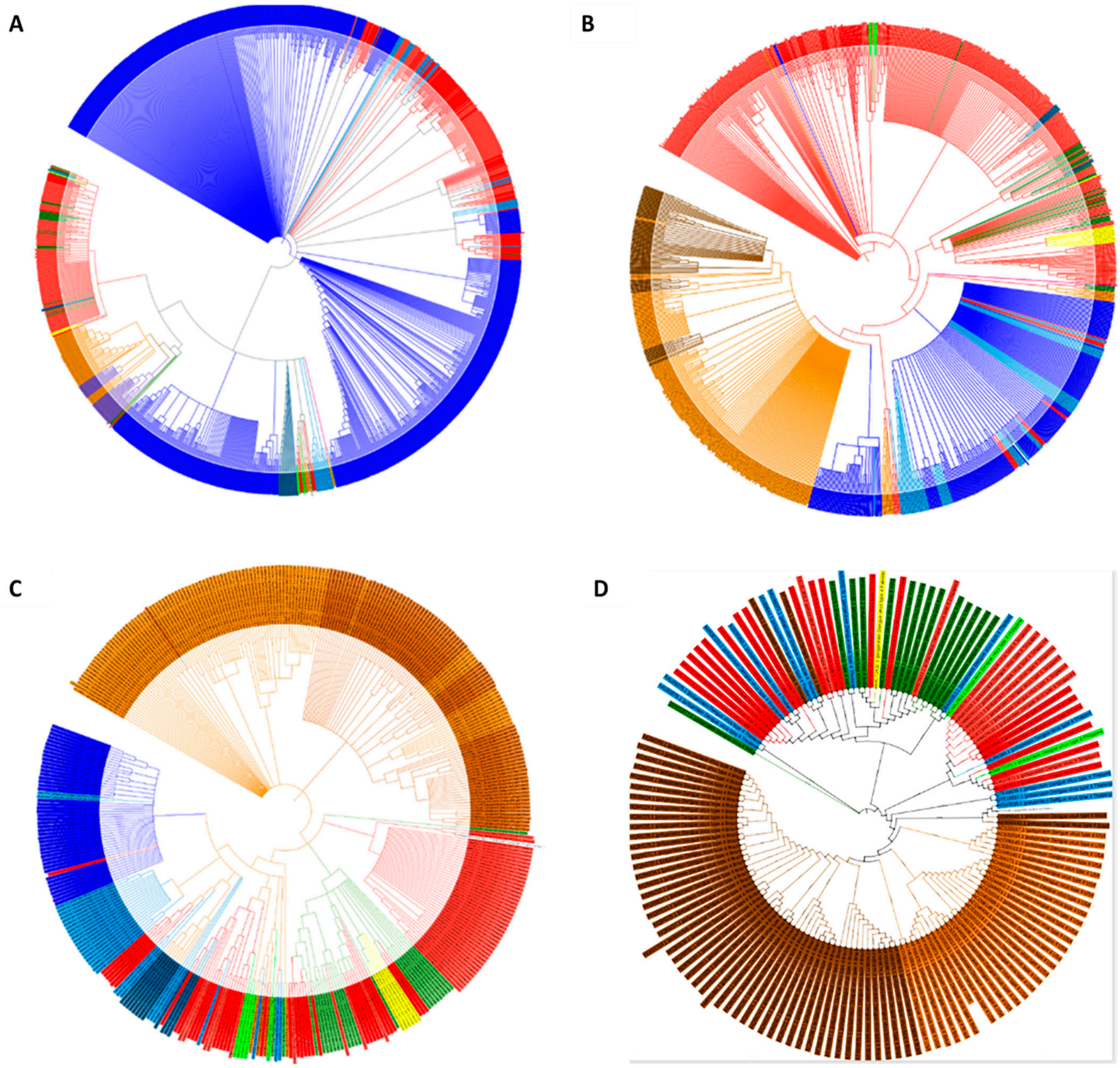

Figure S1. Phylogenetic trees analysis, 10 Countries Combined, (1)Bangladesh,(2)Brazil,(3)China,(4) India,(5)Phillipian,(6) Pakistan, (7)Singapore,(8)Thailand, (9)USA, (10)Vietnam **(A)** Dengue-1 Total Taxa's 1355, 1 Reference strain>NP_059433.1 polyprotein DENV-1, **(B)** Dengue-2 Total Taxa's 1031, 1 Reference strain>NP_056776.2 polyprotein DENV-2, **(C)** Dengue-3 Total Taxa's 400, 1 Reference strain>YP_001621843.1 DENV-3, **(D)** Dengue-4 Total Taxa's 143,1 Reference strain>NP_073286.1polyprotein DENV-4.

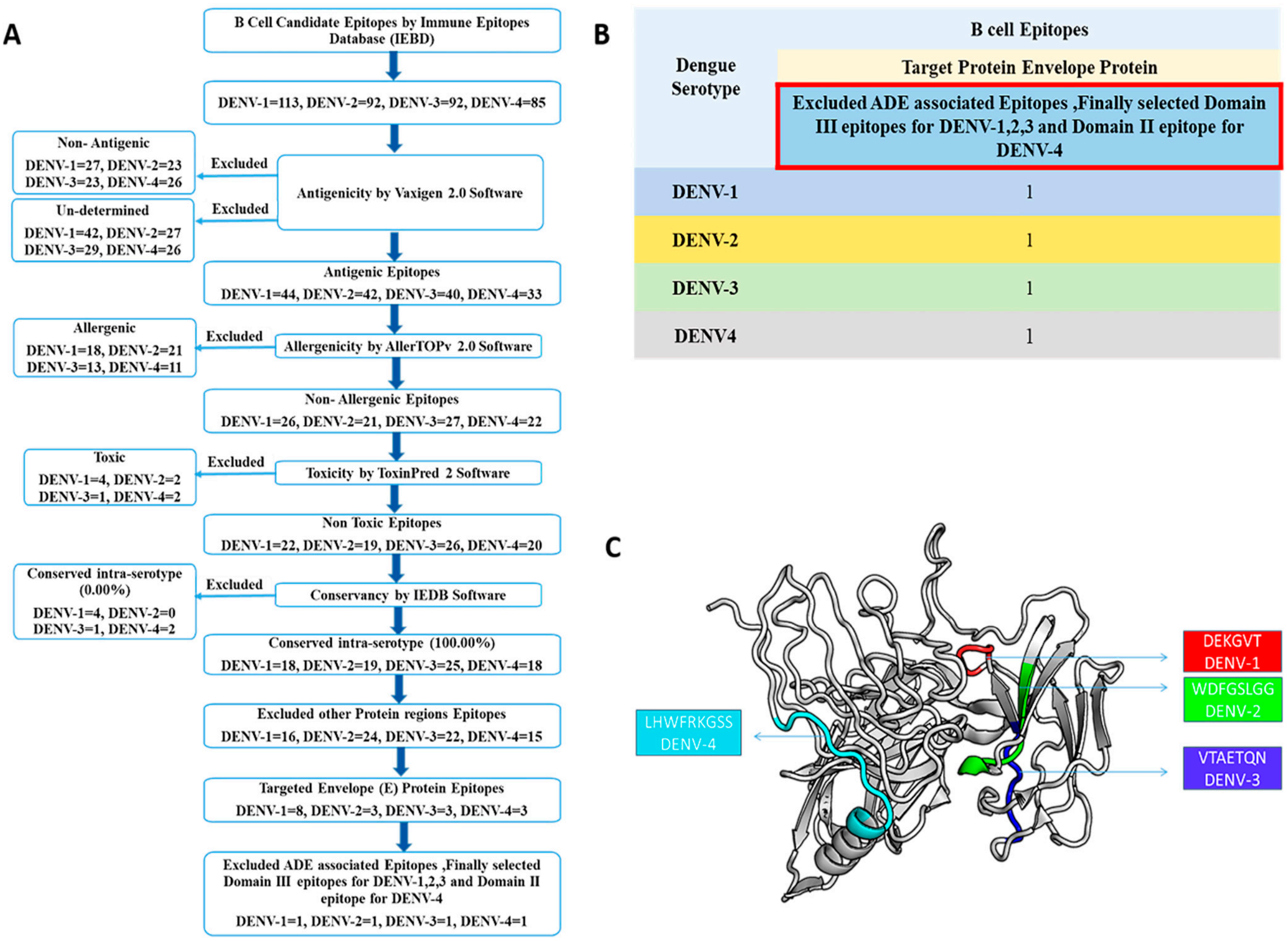

Figure S2. B cell Epitopes, **(A)** Stepwise Selection Procedure and the resultant B cell Epitopes, **(B)** Envelope Protein Epitopes of each serotype, **(C)** DENV1~4 Epitopes Exposed to Envelope Protein surface.

A

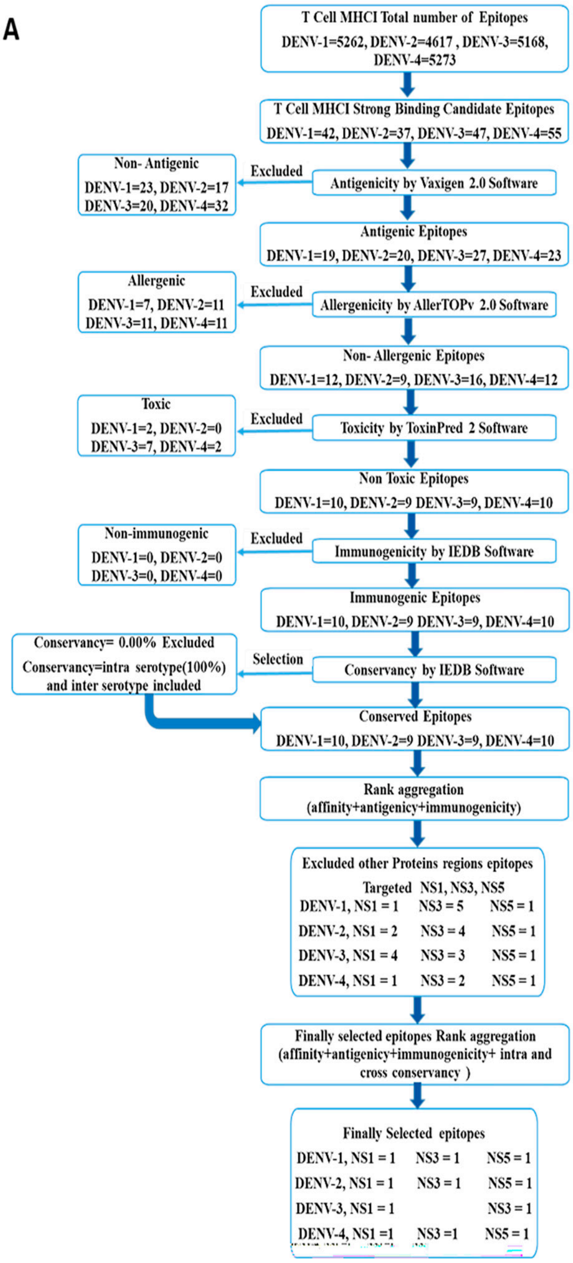

B

Dengue Serotype	MHC-I T cell Epitopes Finally selected epitopes Rank aggregation (affinity+antigenicy+immunogenicity+ intra and cross conservancy)		
	Non- Structural		
	NS1	NS3	NS5
DENV-1	1	5	1
DENV-2	2	4	1
DENV-3	4	3	1
DENV4	1	2	1

C

Dengue Serotype	MHC-I T cell Epitopes Finally selected epitopes in vaccine peptide designing		
	NS1	NS3	NS5
DENV-1	1	1	1
DENV-2	1	1	1
DENV-3	1	1	Conserved DENV-2
DENV4	1	2	1

Figure S3. T cell MHC-I Epitopes**, (A)** Stepwise Selection Procedure and the resultant MHC-I Epitopes, **(B)** Summary of Targeted Proteins NS1, NS3, NS5 Epitopes Selection strategy, **(C)** finally selected epitopes numbers.

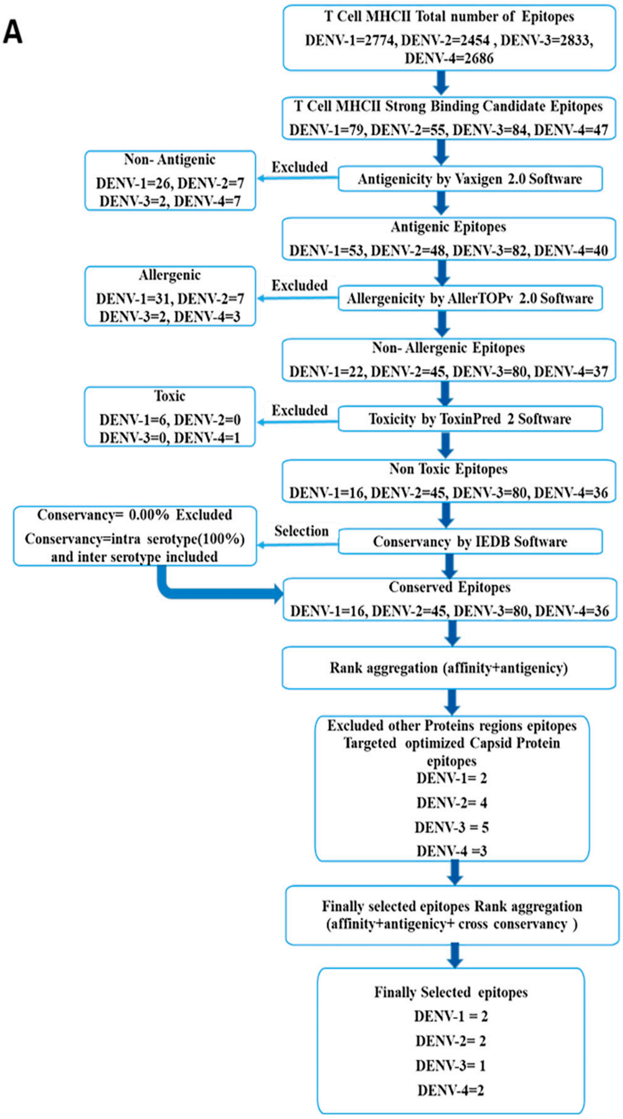

B

Dengue Serotype	Optimized MHC-II T cell Epitopes Targeted Protein Region Rank aggregation (affinity+antigenicy+ intra and cross conservancy)
	Structural Protein
	Capsid (C)
DENV-1	2
DENV-2	4
DENV-3	5
DENV4	3

C

Dengue Serotype	MHC-II T cell Epitopes Finally selected epitopes in vaccine peptide designing
	Capsid (C)
DENV-1	4
DENV-2	1
DENV-3	1
DENV4	1

Figure S4. T cell MHC- II Epitopes**, (A)** Stepwise Selection Procedure and the resultant MHC-II Epitopes**, (B)** Summary of Targeted Capsid Protein Epitopes Selection strategy, **(C)** Finally selected epitope numbers.

A

>PSDV~1 577aa
MAENPNIDDLPAPLLAALGAADLALATVNDLIANLRERAEETRAETRTRVEERRARLTKFQED
LPEQFIELRDKFTTEELRKAAEGYLEAATNRYNELVERGEAALQRLRSQTAFEDASARAEGYV
DQAVELTQEALGTVASQTRAVGERAAKLVGIELEAAAKAKFVAAWTLKAAAGGGSDEKGVTKK
WDFGSLGGKKVTAETQNKKLHWFRKGSSIGGGSFLRFLAIPPTAGVLAGPGPGEIVDLMCHAT
FTMRLGPGPGWCGSLIGLTSRATWAGPGPGRDMTLIMIGSNASDRGPGPGITALILGAQALPV
YLGPGPGQKQSHWVEITALILGGPGPGDFVVTTDISEMGANFMLMTGTLAVAYYLLMRTTWAL
AYYFMNEDHWFSAYYLVAGGLLTVAYYLMMRTTWALAYYLMMRTTWALAYYKLVDRERELAYY
HMIAGVTFVAYYKLNDWDFVVAYYGLLCLTLFVAYYKLTDWDFVVAYYKLTDWDFVVAYYTTA
NWLWALGGGSEAAAKGIINTLQKYYCRVRGGRCAVLSCLPKEEQIGKCSTRGRKCCRRKK

B

>PSDV~2 508aa
MAENPNIDDLPAPLLAALGAADLALATVNDLIANLRERAEETRAETRTRVEERRARLTKFQED
LPEQFIELRDKFTTEELRKAAEGYLEAATNRYNELVERGEAALQRLRSQTAFEDASARAEGYV
DQAVELTQEALGTVASQTRAVGERAAKLVGIELEAAAKAKFVAAWTLKAAAGGGSDEKGVTKK
WDFGSLGGKKVTAETQNKKLHWFRKGSSIGGGSFLRFLAIPPTAGVLAGPGPGEIVDLMCHAT
FTMRLGPGPGWCGSLIGLTSRATWAGPGPGRDMTLIMIGSNASDRGPGPGITALILGAQALPV
YLGPGPGQKQSHWVEITALILGGPGPGDFVVTTDISEMGANFGGGSMLMTGTLAVAYYLLMRT
TWALAYYFMNEDHWFSAYYLVAGGLLTVAYYLMMRTTWALAYYKLVDRERELAYYHMIAGVTF
VAYYKLNDWDFVVAYYGLLCLTLFVAYYKLTDWDFVVAYYTTANWLWALEAAAKAKFVAAWTL
KAAA

C

>PSDV~3 394aa
GIINTLQKYYCRVRGGRCAVLSCLPKEEQIGKCSTRGRKCCRRKKEAAAKAKFVAAWTLKAAA
GGGSMLMTGTLAVAAYLLMRTTWALAYYFMNEDHWFSAYYLVAGGLLTVAYYLMMRTTWALAY
YKLVDRERELAYYHMIAGVTFVAYYKLNDWDFVVAYYGLLCLTLFVAYYKLTDWDFVVAYYTT
ANWLWALGGGSFLRFLAIPPTAGVLAGPGPGEIVDLMCHATFTMRLGPGPGWCGSLIGLTSRA
TWAGPGPGRDMTLIMIGSNASDRGPGPGITALILGAQALPVYLGPGPGQKQSHWVEITALILG
GPGPGDFVVTTDISEMGANFGGGSDEKGVTKKWDFGSLGGKKVTAETQNKKLHWFRKGSSIEA
AAKAKFVAAWTLKAAA

Figure **S5**. Peptide Sequences of constructed, **(A)** PSDV-1, 577 aa Sequences, **(B)** PSDV-2, 508 aa Sequences, **(C)** PSDV-3, 394 aa Sequences

A

Population/ Area	Class Combined		
	Coverage [a]	Average-hit [b]	Pc90 [c]
World	62.26%	5.23	0.53
Average	62.26%	5.23	0.53
Standard Deviation	0.0	0.0	0.0

B

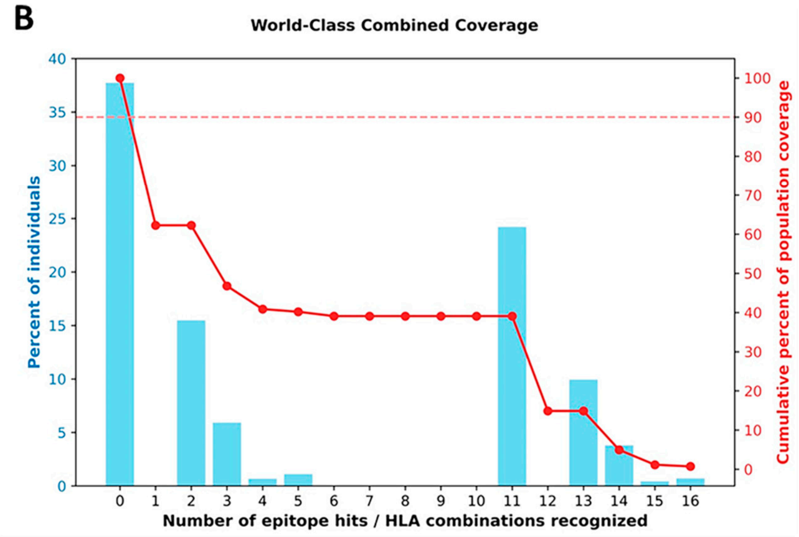

Figure S6. Worldwide Population Coverage, **(A)** MHC-I & MHC-II Classes Combined Average statistics, **(B)** MHC-I & MHC-II Classes Combined Graphically Presentation.

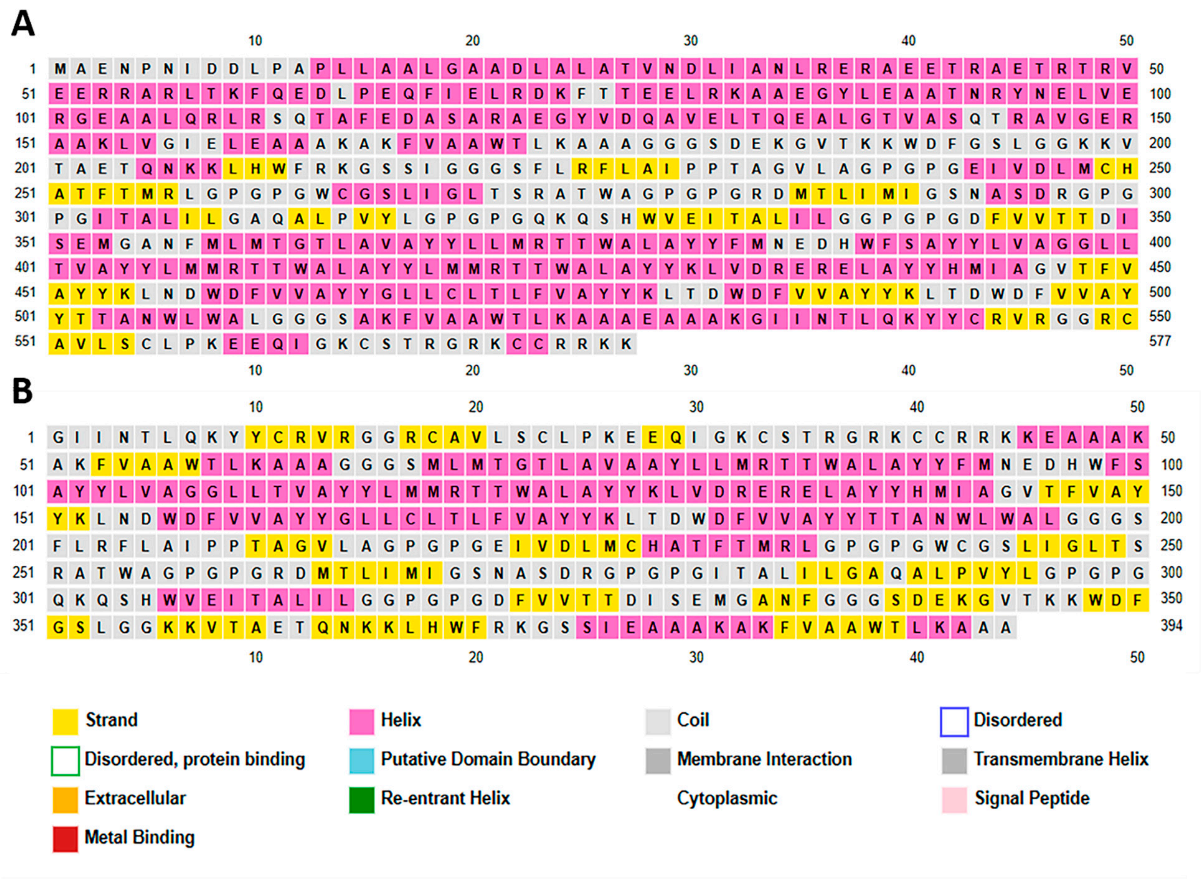

Figure S7. Secondary Structures Generated, **(A)** PSDV-1 vaccine, **(B)** PSDV-3 vaccine.

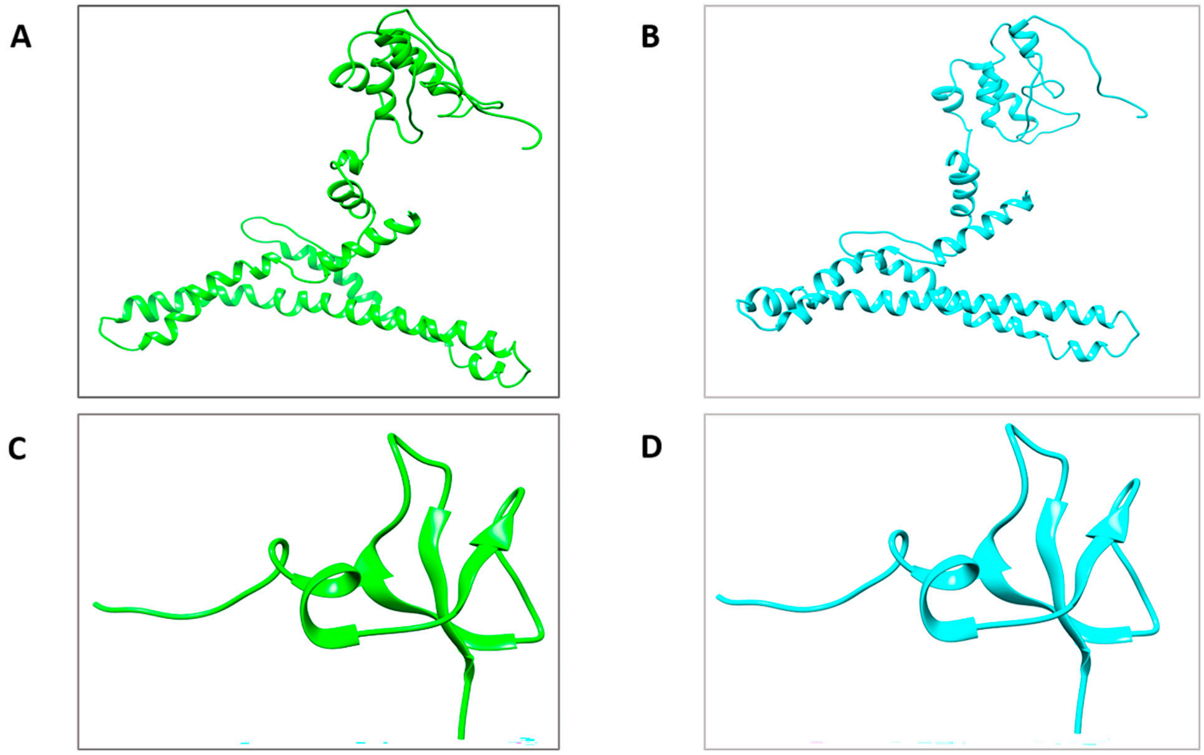

Figure S8. Tertiary Structures Modelled, **(A)** PSDV-1 vaccine model template, **(B)** PSDV-1 vaccine, **(C)** PSDV-3 vaccine model template, **(D)** PSDV-3 vaccine.

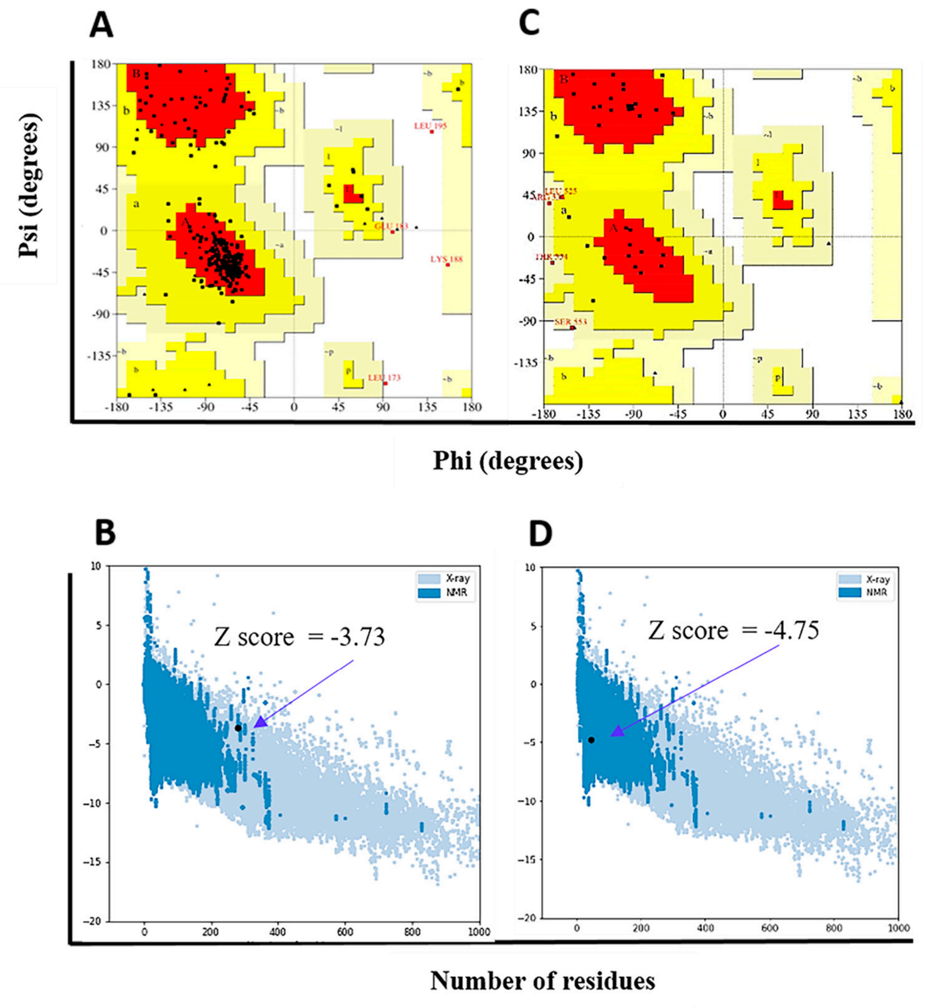

Figure S9. Tertiary Structures validations; Ramachandran **Plots of PSDV1 & 3: This figure displays the Ramachandran plot with annotations for residues in favored, allowed, and outlier regions: (A) PSDV-1, (C) PSDV-2, Z-Score values of PSDV1 &3: Summarizes the Z-scores values of tertiary structures for PSDV1, and PSDV3 (B) PSDV-1, and (D)** PSDV-3.

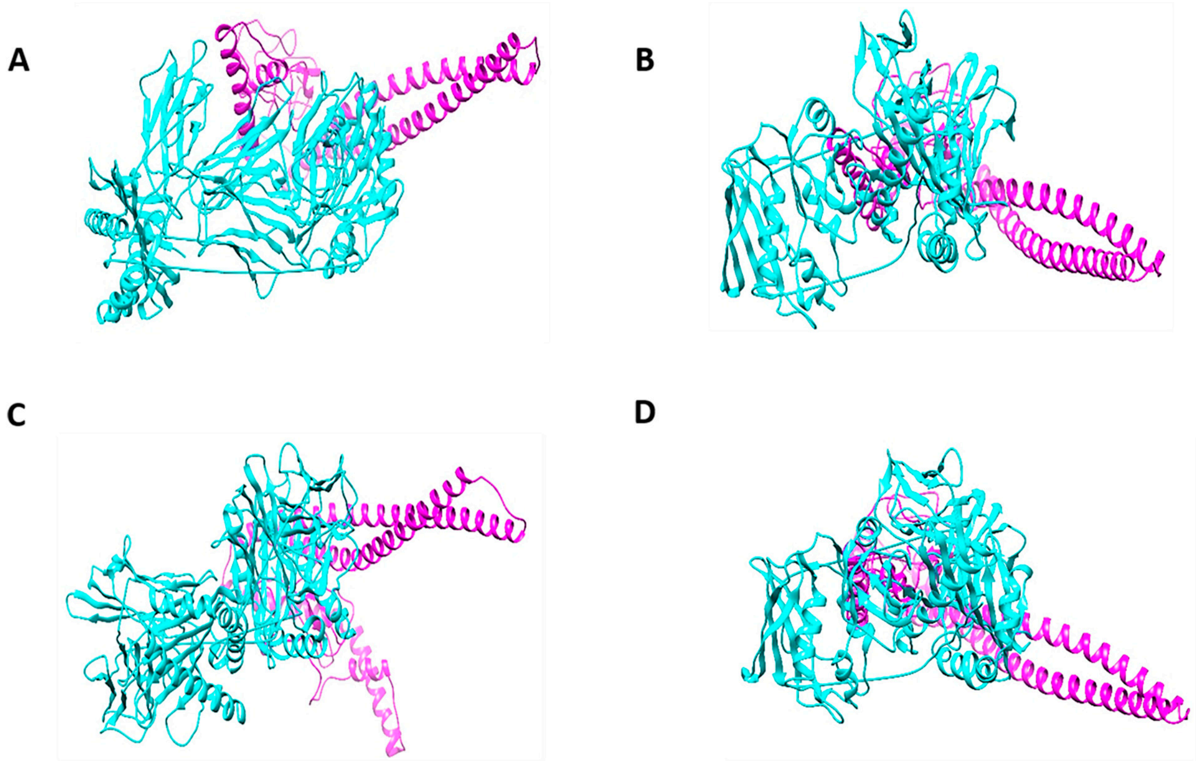

Figure S10. Molecular Docking of PSDV2 Vaccine with Immune Receptors. PSDV2 docking with HLAs molecules **(A)** HLA-DRB-101:01, **(B)** HLA-DRB-104:01, **(C)** HLA-A-0201 and **(D)** HLA-DRB-107:01, respectively.
